# The impact of financial incentives promoting biosimilar products in oncology: A quasi-experimental study using administrative data

**DOI:** 10.1371/journal.pone.0312577

**Published:** 2024-11-14

**Authors:** Hisashi Itoshima, Daisuke Takada, Etsu Goto, Noriko Sasaki, Susumu Kunisawa, Yuichi Imanaka

**Affiliations:** 1 Department of Healthcare Economics and Quality Management, Graduate School of Medicine, Kyoto University, Kyoto, Japan; 2 Department of Health Security System, Centre for Health Security, Graduate School of Medicine, Kyoto University, Kyoto, Japan; University of KwaZulu-Natal, SOUTH AFRICA

## Abstract

**Background:**

Biosimilars have the potential to save a significant amount of money in cancer treatment costs. However, barriers exist in the adoption of biosimilar products. Japan introduced a new health policy in 2022 to promote the use of biosimilars in oncology by offering financial incentives to eligible hospitals. This study aims to examine the association between these financial incentives and prescription patterns.

**Methods:**

The study analyzed Diagnosis Procedure Combination (DPC) data to assess the impact of the new health policy on the use of biosimilar products in oncology. The policy provided an additional fee for hospitals using biosimilar products. The study included patients with specific types of cancer and analyzed the proportion of monthly biosimilar prescriptions using the number of prescriptions of reference and biosimilar products. A generalized synthetic control method was used for analysis.

**Results:**

From April 2020 to March 2023, the study involved 27,737 patients in 114 hospitals, with 63 eligible hospitals receiving financial incentives. The average number of prescriptions of the drugs (rituximab, trastuzumab, and bevacizumab) increased gradually in both eligible and ineligible hospitals. The financial incentives were associated with a significant increase in the proportion of biosimilar product prescriptions, with a monthly increase of 0.092 per month (95% CI, 0.040–0.145) [9.2%, 95% CI, 4.0–14.5] compared to ineligible hospitals.

**Conclusion:**

Our study indicates that providing financial incentives to hospitals to utilize biosimilar products increased their prescriptions. Japan’s recent health policy of moderate financial incentives is an effective approach to increasing prescriptions of biosimilar products.

## Introduction

Based on their effectiveness, biologic products are widely used in clinical practice to treat cancer, collagen vascular disease, and inflammatory bowel disease [1–5]. In 2019, a survey of all cancer drugs approved by the European Medicines Agency (EMA), the US Food and Drug Administration (FDA), and the Japan Pharmaceuticals and Medical Devices Agency (PMDA) found that 27% of new cancer drug approvals were for biologic products [6]. However, biologic products are expensive and place a significant burden on healthcare costs in these countries [6–8]. One cost-saving solution is to use biosimilar products instead of reference products [9,10].

Biosimilars are substances that closely resemble reference biologics in terms of their structure, effectiveness, and safety [11]. Regulatory approval has been granted to three therapeutic oncology biosimilars (bevacizumab, trastuzumab, and rituximab) in the EU, the US, and Japan. The EU, US, and Japan expect to achieve healthcare cost savings of over $110 billion (USD) by 2029, primarily due to substituting oncology biosimilars for reference biologics [6,12]. Therefore, studies promoting the use of biosimilars have been conducted in several countries, revealing various obstructions to their use among medical staff, patients, and medical systems [13–18]. To address these barriers, researchers have reexamined the national pricing system for biosimilar drugs and educated clinicians and patients about the efficacy and safety of biosimilars [6].

The government of Japan introduced the Basic Policy on Economic and Fiscal Management and Reform in 2021. It states that to promote setting targets based on the effectiveness of medical cost optimization for biosimilars, a review of the generic drug prescription system (based on the relationship with the new targets) should be considered to promote further use of these kinds of drugs [19]. In April 2022, the Ministry of Health, Labour, and Welfare (MHLW) in Japan introduced a new health policy to promote using biosimilar products instead of original products in oncology [20]. When commencing chemotherapy using biosimilar products (rituximab, trastuzumab, and bevacizumab) or switching the original drugs to biosimilar products, eligible hospitals might claim an additional award (1,500 Japanese Yen (JPY) per patient per month) for up to three months [20]. However, changes in prescription patterns have not been examined before and after the introduction of the new health policy. We hypothesized that the new health policy would lead to an increase in the use of biosimilar products instead of reference products.

The current study aimed to identify an association between financial incentives promoting biosimilar products rather than reference products in oncology and changes in prescription patterns using Japanese administrative data.

## Materials and methods

### Data source and collection

We performed a quasi-experimental, generalized synthetic control (GSC) study using Diagnosis Procedure Combination (DPC) data from the Quality Indicator/Improvement Project (QIP) database, which is managed by the Department of Healthcare Economics and Quality Management, Kyoto University. The QIP database comprises DPC data from acute care hospitals that have willingly chosen to participate in the project. The program encompasses more than 500 hospitals, both public and private institutions, that are distributed across Japan [21].

The DPC/pre-diem payment system (PDPS) is a prospective payment system utilized in acute care hospitals in Japan. In 2020, the DPC/PDPS system was implemented in 1,757 hospitals, collectively having 483,180 beds. This figure encompasses almost all admissions for short-term hospital stays and approximately 54% of all beds in hospitals with general beds across the country [22]. The DPC data consist of clinical summary information, insurance claims, facility identifiers, admission and discharge statuses (live discharge or in-hospital death), primary diagnosis, the most and second-most resource-intensive diagnoses, up to ten comorbidities, and up to ten complications. The DPC data also includes medical charges, implementation dates, and the frequency and quantity of medical treatments (e.g., procedures and tests), as well as prescription drugs and medical supplies [22]. Additional information has recently been published [22]. We accessed the database for this study from August 21, 2023 to November 20, 2023, and didn’t access to information that could identify individual participants during or after data collection.

### Quasi-experiment: Japanese new health policy

The new health policy (financial incentive: The fee for the initial introduction of biosimilars) was introduced on April 1, 2022, as part of the push to use biosimilar medications in place of original products in oncology. This policy was based on the Basic Policy on Economic and Fiscal Management and Reform 2021 [19]. Eligible hospitals may receive an additional fee of 1,500 JPY (≈ 10.4 US dollars: USD, 9.5 Euro: EUR) per patient per month for up to three months (i.e., eligible hospitals could get an additional fee of 4,500 JPY maximum (≈ 31.2 USD, 28.4 EUR) per patient) when using biosimilar products (rituximab, trastuzumab, and bevacizumab) to start chemotherapy or when switching the original drugs to biosimilar products [20]. In order to qualify for this incentive, the patient must be informed of the biosimilar products’ safety and efficacy compared to their originators when a prescription is written.

As a foundation of the new policy, the Outpatient Oncology Chemotherapy Management Fee aims to create a collaborative treatment system involving doctors, nurses, pharmacists, and other healthcare professionals. A range of criteria must be met for a hospital to be eligible for this fee, including a bed set aside for treatment, emergency hospitalization facilities in case a patient becomes ill while receiving treatment, a full-time physician, nurse, or pharmacist with specialty staff on at all times, a 24-hour contact system set up to answer questions from patients, and information about the benefits and side effects of chemotherapy provided to patients [20]. The cost of the three biosimilar products was about 40%–60% cheaper compared to the original products, and their details are described in S1 Table. Hospital eligibility was determined based on whether the financial incentive (the fee for the initial introduction of biosimilars) was calculated.

### Study population

The International Classification of Diseases, Tenth Revision (ICD-10) codes were used to categorize all diagnoses. Using the DPC data from April 2020 to March 2023, we included inpatients with a primary disease identified as gastric cancer, colorectal cancer, lung cancer, breast cancer, B-cell lymphoma, or B-cell proliferative disease who received chemotherapy (rituximab, trastuzumab, bevacizumab). Since the DPC data has no summary information for outpatients, we included those with ICD10 codes for gastric cancer, colorectal cancer, lung cancer, breast cancer, B-cell lymphoma, or B-cell proliferative disease, extracted from the EF file, who were treated with the corresponding chemotherapy. Finally, only cases from hospitals with data for all 39 months were included in this study. The ICD-10 codes we used are described in S2 Table.

### Outcomes of interest

In Japan, the adoption of generic drugs often involves switching from brand-name drugs, and the same drugs tend to be used for inpatients, in-hospital outpatients, and out-of-hospital uses (i.e., when a generic drug is adopted, the brand-name drug with the same effect is excluded from the drugs adopted in the hospital). Therefore, we assumed that prescriptions of biosimilar products increased and those of reference products decreased due to the new health policy.

Proposed by the World Health Organization (WHO), the Defined Daily Dose (DDD) is often used to compare drug use internationally; however, the DDD of antineoplastic products (ATC group L01) has not been reported [23]. In addition, the data used in this study contained scattered outliers related to drug usage. Therefore, our primary outcome was to compare the proportion of monthly prescriptions for biosimilar products with prescriptions for reference products. Thus, we calculated the monthly proportion of prescriptions for biosimilar products using the number of prescriptions for biosimilar products and reference products as the denominator and the number of prescriptions for biosimilar products as the numerator. Our secondary outcome was the monthly costs of biosimilar and reference products.

### Covariates

The Japanese government implemented the National Cancer Control Act in 2007, and based on this law, the Basic Plan to Promote Cancer Control Programs has been promoted. Consequently, the MHLW has identified about 400 hospitals as Designated Cancer Care Hospitals (DCCHs) [24,25]. These hospitals must meet national criteria regarding the number of surgeries for cancer patients, medical staff expertise, and support programs for cancer patients. If the DDCHs receive cancer patients, they could receive a financial incentive, “the fee for DCCHs.” Since a hospital must have a system in place to treat cancer patients to qualify for the “fee for the initial introduction of biosimilars,” DDCHs were considered more likely to receive this award. Therefore, we decided to include the presence or absence of the fee for DCCHs as an adjustable variable in the statistical analysis model.

### Statistical analyses

#### Quasi-experimental design

Quasi-experiments evaluate events or interventions that are difficult or impossible to manipulate experimentally, such as many policy and healthcare reforms [26]. The study designs of quasi-experiments include interactive fixed effects (IFE), difference-in-differences (DID), interrupted time series (ITS), synthetic control methods (SCMs), and generalized SCMs [27,28]. Nianogo et al. found that data adaptation methods such as generalized SCMs (GSCM) were usually less biased than the other methods evaluated in their study when there were data from multiple time points and multiple control groups (multigroup design) [28].

Xu et al. proposed GSCMs to integrate an interactive fixed effects model with synthetic controls [29]. This method can be applied when time-series cross-sectional data are available, and it is likely that the concurrent trend assumptions, variable treatment periods, and multiple treatment units won’t hold true. Generalized synthetic control techniques can use data from control groups to impute counterfactual outcomes for every treatment unit to estimate the average treatment effect (ATT) across treatments [29,30]. Time variables, time-delayed outcomes (i.e., outcomes of interest during the pre-exposure period), and time-varying covariates are used to decompose the control group time series. Next, for each treatment unit, a weighting procedure is used to determine which composite control group is most appropriate (during the pre-treatment phase). Then, a fictitious trend in the treatment group (in the absence of treatment) is estimated using this data. This approach is comparable to the conventional synthetic control method, employing a reweighting procedure to determine the appropriate counterfactual virtual group by weighting the control units based on the pre-treatment results [29,30]. In GSCM, standard error estimates are calculated using bootstrapping techniques—we conducted 500 bootstrapping iterations.

We assumed that the new reimbursement system, introduced in April 2022, would not affect eligible facilities immediately since the system would need to be in place before it could be obtained. Each facility would obtain this addition after its system was ready. As such, the timing of the exposure factor effect would differ for each facility.

Sensitivity analysis was conducted using DID. We set the intervention time to April 2022, when the Japanese health policy changed for all hospitals. In addition, we analyzed the impact of the new health policy (financial incentive) for each drug using GSCM. Details of our models are described in S3 Table.

We conducted these analyses using the gsynth package (version 1.2.1) for R [31]. A two-sided P-value less than 0.05 was deemed statistically significant, and all analyses were performed using R 4.2.2 (R Foundation for Statistical Computing, Vienna, Austria). The study was conducted in compliance with the appropriate institutional guidelines and regulations.

### Ethical statement

The utilization of anonymized data allowed us to waive the need for informed consent, as per the Ethical Guidelines for Medical and Health Research Involving Human Subjects (a provisional translation can be found at: https://www.mhlw.go.jp/file/06-Seisakujouhou-10600000-Daijinkanboukouseikagakuka/0000080278.pdf), as mandated by the Japanese government. As per Part 12, sections (1), (2), and (6) of these Guidelines, researchers have the option to waive the requirement of informed consent for a study that makes use of pre-existing information. The current study received approval from the Ethics Committee at the Graduate School of Medicine, Kyoto University. The committee waived the requirement for informed consent for this study (approval number: R0135). The methods were executed in compliance with the applicable guidelines and regulations.

## Results

We included 27,737 patients in 114 hospitals during the study period. Sixty-three hospitals were eligible for financial incentives. The characteristics of each hospital are described in Table 1; eligible hospitals had more beds and received more patients compared with ineligible hospitals.

**Table 1:** The characteristics of eligible and ineligible hospitals.

Hospitals, N = 114	Eligible	Ineligible
	N = 63	N = 51
Administrator, N		
Public	40	23
Private	21	26
Missing	2	2
Hospital beds, N		
Median, IQR	424 (350, 600)	264 (248, 425)
200 > =	2	8
400 > = ~ 200>	21	25
400>	38	16
Missing	2	2
Designated cancer care hospital*, N	37	12
Number of patients, N		
Inpatients, N	10,520	4,049
Outpatients, N	9,269	3,899
Cancer type (Patients), N		
Lung	2,186	955
Colorectal	2,815	1,616
Gastric	543	258
Breast	2,713	1,261
B cell	8,749	1,934
Unknown**	2,783	1,924

IQR, inter quantile range.

* A designated cancer care hospital appointed by the Japanese government has to meet national criteria regarding the number of surgeries for cancer patients, medical staff expertise, and support programs for cancer patients.

**Patients with several diagnoses were not classified into any category.

**Fig 1:**
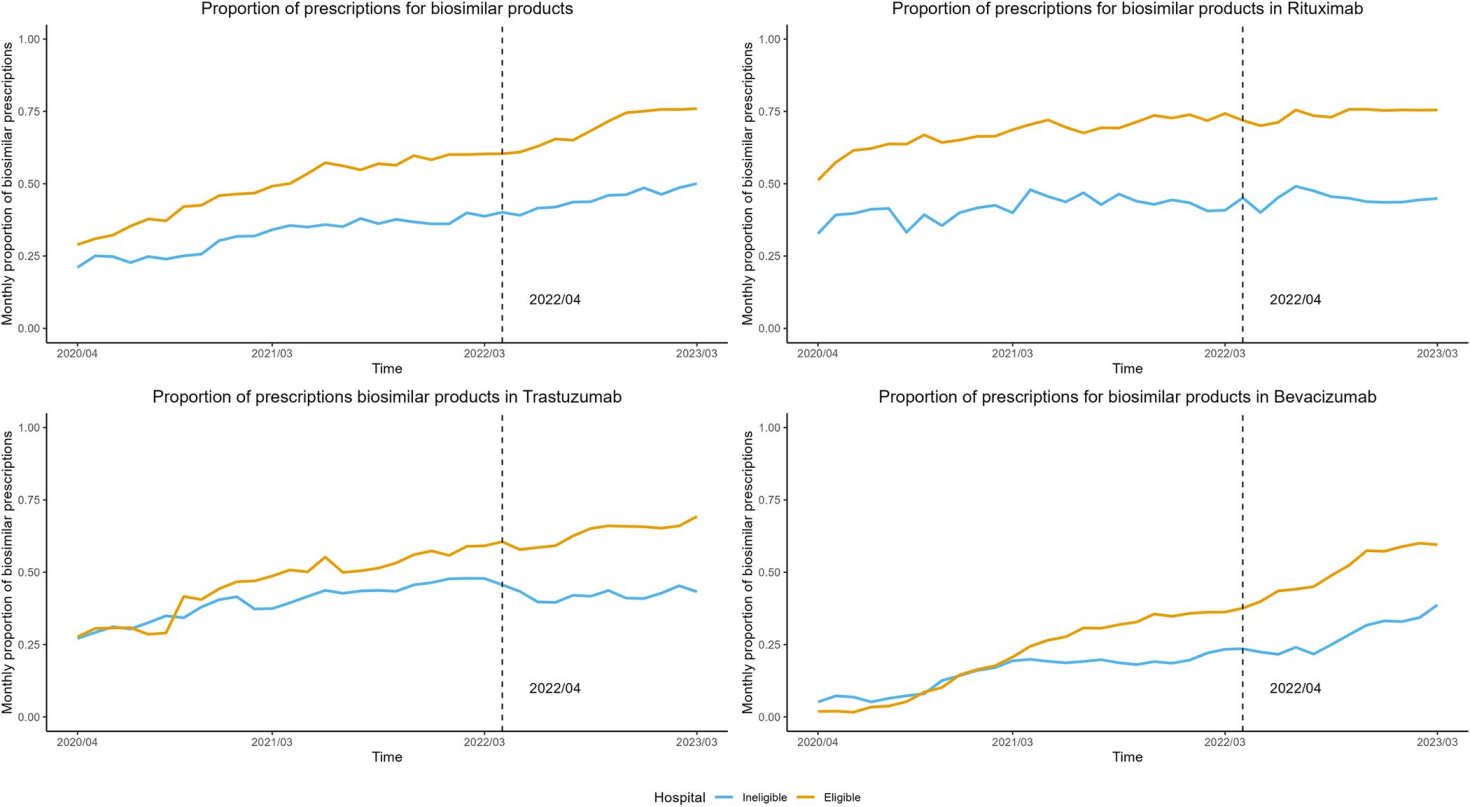
The monthly proportion of biosimilar products’ prescriptions between eligible and ineligible hospitals. The average proportion of biosimilar product prescriptions (rituximab, trastuzumab, and bevacizumab) by hospital type. The financial incentive for promoting biosimilar products was introduced in April 2022.

In both eligible and ineligible hospitals, the average proportion of overall prescriptions for biosimilar products (rituximab, trastuzumab, and bevacizumab) increased gradually (Fig 1). The number of biosimilar prescriptions by drug type increased, while reference products decreased in both hospital types (S1 Fig). S2 Fig illustrates the implementation status of the new health policy (financial incentive) in each hospital, revealing a variation in the timing of this policy’s effect across hospitals.

### The monthly proportion of biosimilar products’ prescription

**Fig 2:**
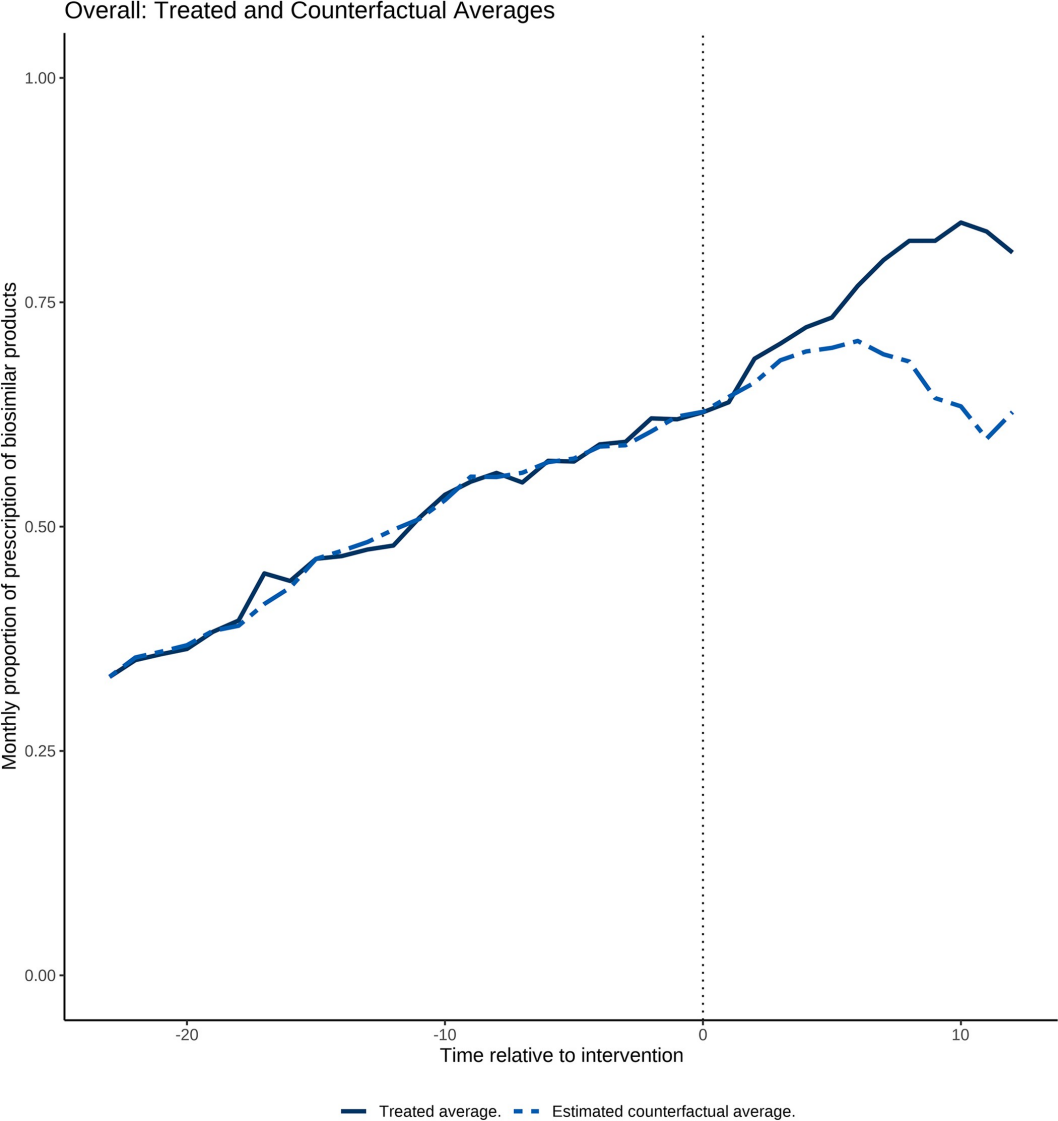
The difference between treated and estimated counterfactual monthly proportion of prescriptions of biosimilar products by month. The difference between the treated (solid) and counterfactual (dashed) lines represents the beta estimate of the financial incentive (the new health policy). The financial incentive to promote the use of biosimilar products was introduced in April 2022 (corresponding to 0 on the X-axis).

**Fig 3:**
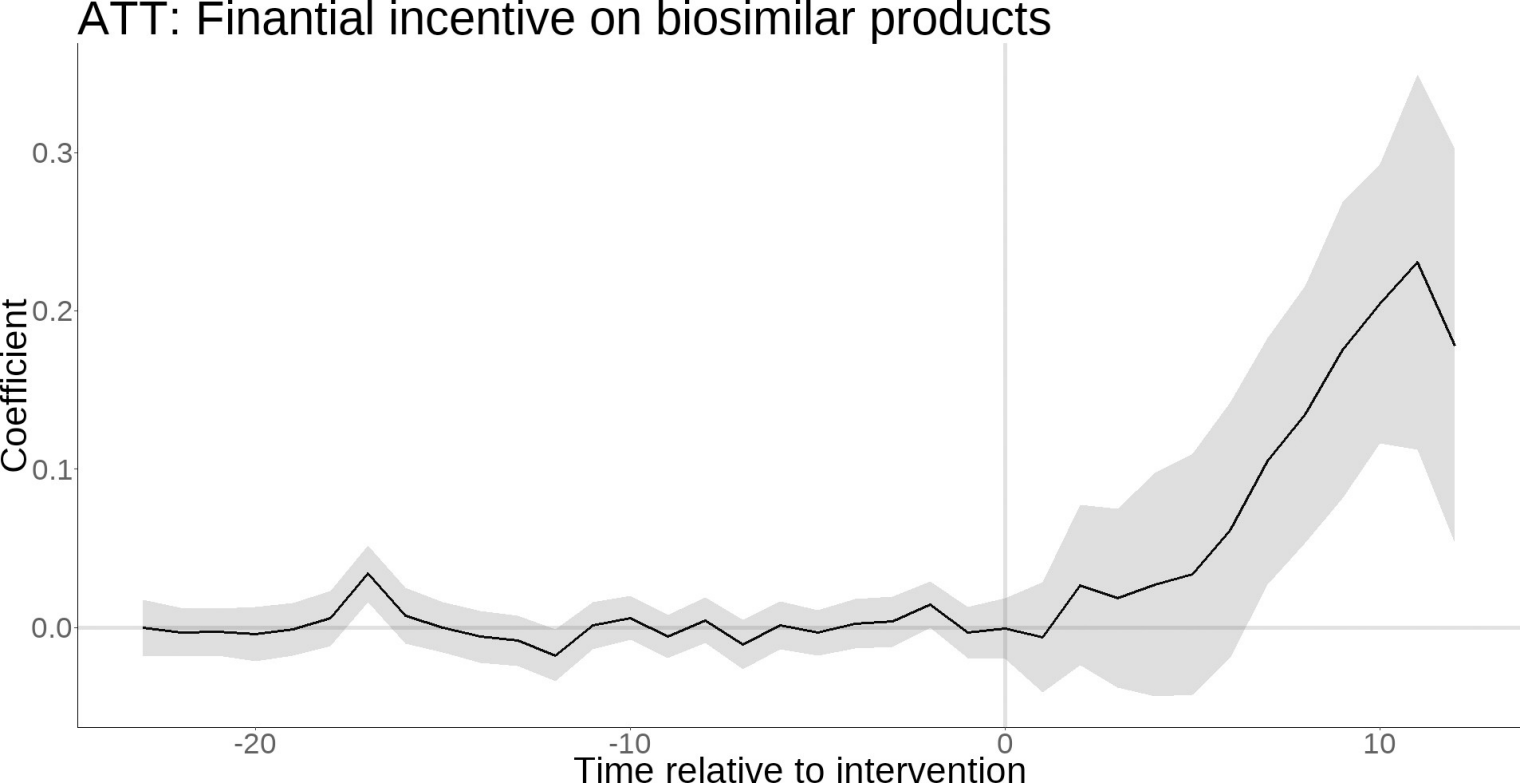
The average treatment effect of the financial incentives for biosimilar products. This figure displays the coefficients on the Y-axis (monthly proportion of prescriptions), the time relative to treatment on the X-axis, and the 95% confidence intervals in the shaded region. The financial incentive to promote the use of biosimilar products was introduced in April 2022 (corresponding to 0 on the X-axis).

The GSCM analysis demonstrated that financial incentives were related to an increase in the proportion of biosimilar product prescriptions, 0.092 per month (95% CI, 0.040–0.145) [9.2%, 95% CI, 4.0–14.5], compared with the ineligible hospitals (Figs 2 and 3 and Table 2). The results of the sensitivity analysis using DID, in which the intervention time was considered to be April 2022 for all target hospitals, also showed similar results to the main analysis (proportion of biosimilar product prescriptions: 0.081 per month (95% CI, 0.001–0.163) [8.1%, 95% CI, 1.0–16.3].

**Table 2:** Estimates of the impact of financial incentives for using biosimilar products.

	ATT	95% CI	p-value
Primary outcome: Monthly proportion of biosimilar products’ prescription			
Generalized synthetic control method (GSCM)*			
Biosimilar products	0.092	(0.040 to 0.145)	0.001
Difference in differences (DID)**			
Biosimilar products	0.081	(0.001 to 0.163)	< 0.001
Secondary outcome: Expenditure for biosimilar and reference products (1,000 JPY)			
GSCM*	1872.958	(-120.918 to 3866.835)	0.56
DID**	1146.7	(-1835.508 to 4128.908)	0.16

ATT: Average treatment effect on treated, CI: Confidence interval, JPY: Japanese Yen.

*Standard error estimates were calculated using bootstrapping techniques, we conducted 500 bootstrapping.

**Hospitals were setted to fixed effect in our model.

### Monthly expenditure summed for biosimilar products and reference products

The average monthly expenditure summed for biosimilar products and reference products (rituximab, trastuzumab, and bevacizumab) in both eligible and ineligible hospitals decreased gradually (S3 Fig). The secondary outcome of monthly expenditure was not significantly associated with financial incentives; the impact of the incentive was 1872.958 (1,000 JPY) (95% CI: -120.918 to 3866.835). The result is shown in S3 Fig.

## Discussion

Biologics are increasingly being used in oncology treatment because of their usefulness, but they are expensive and heavily burden healthcare costs. Biosimilar products, closely resembling reference biologics, could help to reduce costs. While there are various barriers to expanding the use of biosimilar products, the Japanese government has developed a new health policy where hospitals can receive financial reimbursement for their use. In our quasi-experimental study using administrative claims data, we found that the financial incentive (maximum 4,500 JPY per case) was related to an increase in the proportion of biosimilar product prescriptions, 0.092 per month (95% CI, 0.040 to 0.145) [9.2%, 95%CI, 4.0 to 14.5].

A recent systematic review and meta-analyses of the medications included in this study showed no significant difference in efficacy or safety between the reference products and their biosimilar products [32]. The study found that bevacizumab, rituximab, and trastuzumab were comparable to the reference products in terms of the objective response rate, as indicated by the risk ratios and confidence intervals from 16, 12, and 9 randomized controlled trials, respectively (bevacizumab, risk ratio [RR], 0.97, 95% CI, 0.93 to 1.01; rituximab, RR, 1.03, 95% CI, 0.98 to 1.08; trastuzumab, RR, 1.04, 95% CI, 0.97 to 1.12).

Despite these indicators of the usefulness and safety of biosimilars, various obstacles to expanding their use still exist. For example, health policies, interests among the parties involved (such as pricing and rebate payments from pharmaceutical companies), knowledge gaps among healthcare providers, and patient concerns about safety have been reported as obstacles [6,15,33,34]. Regarding health policies, there are three types of policies to promote the use of biosimilars: tendering, healthcare professional incentives, and pharmacist-led substitution [35]. Tendering is a type of tender affecting hospital, regional, and national tenders. For example, Norway and Denmark implement national tendering systems, which help save costs. Healthcare professional incentives are a type of incentive that affects prescription quotas, gain-share agreements, and guidance. Quotas are implemented in Germany, Hungary, Italy, and Sweden. Local reinvestment of savings from the use of biosimilar medicines is facilitated through gain-share agreements. Guidance includes positive recommendations from medical societies and practical advice on implementing biosimilars [35]. Regarding knowledge gaps among healthcare providers, a systematic review assessing the need to educate healthcare providers indicated that US and EU healthcare providers are cautious about the use of biosimilar products, citing poor knowledge of biosimilars and concerns about safety and efficacy as the main disincentives for their use [36]. That review also highlighted the importance of educating healthcare providers about biosimilar products. Patient education is also important. A recent statement on biosimilars from the American Society of Clinical Oncology policy says that clinicians must provide enough information about safety and efficacy for their patients [37].

In Japan, the Basic Policy on Economic and Fiscal Management and Reform 2017 first mentioned promoting the use of biosimilars, and the Basic Policy on Economic and Fiscal Management and Reform 2019 states that the government will promote research, development, and dissemination of biosimilars while keeping track of their efficacy and safety [38]. Based on these plans, MHLW first introduced a financial incentive for using biosimilar products in the 2020 revision of medical fees. The drugs covered by this incentive included insulin, adalimumab, etanercept, teriparatide, and human growth hormone [39]. Further promotion of biosimilars will optimize medical costs, and three drugs used in oncology (rituximab, trastuzumab, and bevacizumab) were added to the list of drugs eligible for additional biosimilar payments in the 2022 revision of medical fees. In Japan, the official price of these drugs is set at about 50%– 60% of the reference product price. This policy is an economic incentive that can be obtained by hospitals having the facilities and human resources to provide anticancer treatment and by explaining the efficacy and safety of biosimilars to patients and gaining their understanding of the drug’s use. The policy not only offers financial incentives to hospitals but also takes patient education into account, which is one of the barriers to expanding the use of biosimilar products.

In this study, the financial incentives did not significantly impact the secondary outcome: drug costs. This may be because, as shown in Figs 1 and S1, prescriptions for reference drugs decreased, and prescriptions of biosimilars increased, but overall, prescriptions for biologic drugs increased, and an overall impact on drug costs did not appear. However, considering that the dose of biosimilar prescriptions is rising in place of the reference products and that the drug price is approximately 40%–60% of the cost of the reference products (e.g., Price of bevacizumab 400 mg; reference 121,608 JPY (≈ 843.4 USD, 766.1 EUR), biosimilar 54,403 JPY (≈ 377.3 USD, 342.7 EUR)), the policy actually reduces costs more than the cost of the financial incentive (Maximum 4,500 JPY (≈ 31.2 USD, 28.4 EUR) per patient).

This study has several limitations. First, there might be unobserved confounding factors and model misspecification. However, the GSCM allows for the control of time-varying covariates and mitigates the parallel trend assumption, which can threaten DID [28,29]. Reportedly, GSCM also presents reliable estimates under various simulations compared to DID, synthetic controls, and interactive fixed effects models [28]. Sensitivity analysis using DID showed similar results to the main analysis. Violations of the common shock assumption threaten the validity of the GSCM results as well as in other quasi-experimental designs. This violation can be determined by other events or policies during the targeted intervention, but it is usually difficult to verify. Therefore, if a hospital introduced an intervention promoting biosimilars during the same period, it could weaken the effect of the new health policy. At least on a national scale, as far as we were able to verify, no measures encouraging greater use of biosimilars were in place during the period when the new health policy was introduced. A previous study reported that physician concerns about safety and efficacy may influence the prescription of biosimilars [36], and the unstable supply of generic medicines in Japan could potentially impact prescription patterns [40]. These factors may have been unmeasured confounders as they were not included in the present study.

Second, patients must be informed of the efficacy and safety of the drug so that the hospital can receive the incentive, but the specifics are unclear. Therefore, patient education content may vary from hospital to hospital, and it may be desirable to detail specific explanations of the conditions for obtaining incentives in the future.

Third, the study sample was limited to hospitals involved in a quality improvement project, which potentially did not give a complete representation of the national healthcare system. Fourth, we did not evaluate clinical outcomes or patient-reported measures. A recent systematic review and meta-analyses of the medications under investigation in this study revealed no notable disparity in effectiveness or safety between the reference products and their biosimilar counterparts [32]. Nevertheless, it is important to consider patient-reported outcomes, such as anxiety associated with the use of biosimilar products. In a future study, examining the relationship between introducing the financial incentive and patient-reported outcomes may be desirable.

Finally, it is difficult to determine the favorable amount of the financial incentives uniformly. Therefore, it is necessary to consider whether current incentives need to increase or whether smaller amounts would be acceptable as incentives and still promote an increase in the use of biosimilars. Currently, the MHLW plans to revise its medical fees in 2024. While the incentive amount is yet to be determined, there are discussions about expanding the number of drugs covered and allowing patients to earn additional benefits while hospitalized [41].

## Conclusions

Our study indicates that providing financial incentives to hospitals to utilize biosimilar products increased their prescriptions. Japan’s recent health policy suggests a potentially effective approach to increasing the use of biosimilars through moderate financial incentives.

## Supporting information

S1 Table The dose and price list of rituximab, trastuzumab and bevacizumab. (DOCX)

S2 Table The ICD-10 codes used in this study. (DOCX)

S3 Table Statistical models of the generalized synthetic control method and difference-in-differences estimates. i = hospitals. T = time period (T0, before the new health policy (financial incentive) was implemented; T1, after the new health policy was implemented); A = treatment variable (A = 0 if the unit did not receive the policy, A = 1 if the unit received the policy); A*T = the average treatment effect on the treated (interaction between the treatment indicator and the time indicator); C = set of unit-time-varying covariates that affect (and not affected by) the outcome and which represents the set of covariates sufficient for confounding control; μ = time-invariant unobserved confounders; λ = time-varying effects that are assumed to be the same for the treated and control units. X_1i_ = intervention at hospitals (0 = no incentive [ineligible], 1 = incentive [eligible]); X_2i_ = time indicator (0 = pre-intervention, 1 = post-intervention); *X_it_* = a covariate that can vary across units i and time t; β0 = constant; β1 = treatment group-specific effect; β2 = time trend common to the eligible and ineligible hospitals; β3 = difference-in-differences estimates.
(DOCX)

S1 Fig The trends in monthly number of prescriptions of biosimilar products and reference products. The average number of prescriptions for biosimilar (rituximab, trastuzumab, bevacizumab) and reference products by hospital.
(TIF)

S2 Fig The status of introducing the new health policy (financial incentive) in each hospital. The y-axis represents each hospital, and the x-axis represents each month from April 2020 to March 2023. Light blue represents hospitals that did not receive financial incentives during the study period. Hospitals with financial incentives are represented in dark blue, while a darker blue indicates when these incentives were obtained.
(TIF)

S3 Fig Trends in the average expenditure for biological products in both eligible and ineligible hospitals. The average overall expenditure on drugs (rituximab, trastuzumab, and bevacizumab) in both eligible and ineligible hospitals.
(TIF)
