# Supplementary material for: The impact of financial incentives promoting biosimilar products in oncology: A quasi-experimental study using administrative data
Source: PLoS One. 2024 Nov 14;19(11):e0312577. doi: 10.1371/journal.pone.0312577 (PMC11563361; doi:10.1371/journal.pone.0312577)
Supplement: S1 Table — (DOCX) [file pone.0312577.s001.docx]

**Supporting information**

Supplement to: Itoshima H, Takada D, Goto E, Sasaki N, Kunisawa S, Imanaka Y.

The impact of financial incentives for promotion of using biosimilar products on oncology field: A quasi-experiment design in administrative data

**Contents**

S1 Table. The dose and price list of rituximab, trastuzumab and bevacizumab

**S1 Table. The dose and price list of rituximab, trastuzumab and bevacizumab**

| Type | Drug name | Dose | Price (JPY) | (USD) | (EUR) |
| --- | --- | --- | --- | --- | --- |
| Reference | Rituxan Intravenous Infusion | 100mg | 24,221 | 168.5 | 152.6 |
| Reference | Rituxan Intravenous Infusion | 500mg | 118,714 | 825.9 | 747.9 |
| Biosimilar | Rituximab BS Intravenous Infusion[KHK] | 100mg | 16,187 | 112.6 | 103.6 |
| Biosimilar | Rituximab BS Intravenous Infusion[KHK] | 500mg | 79,151 | 550.7 | 506.6 |
| Biosimilar | RITUXIMAB BS Intravenous Infusion[Pfizer] | 100mg | 16,187 | 112.6 | 103.6 |
| Biosimilar | RITUXIMAB BS Intravenous Infusion[Pfizer] | 500mg | 79,151 | 550.7 | 506.6 |
|  |  |  |  |  |  |
| Reference | HERCEPTIN for Intravenous Infusion | 60mg | 15,090 | 105 | 96.6 |
| Reference | HERCEPTIN for Intravenous Infusion | 150mg | 34,670 | 241.2 | 221.9 |
| Biosimilar | Trastuzumab BS for I.V. Infusion「NK」 | 60mg | 8,424 | 58.6 | 53.9 |
| Biosimilar | Trastuzumab BS for I.V. Infusion「NK」 | 150mg | 19,118 | 133 | 122.4 |
| Biosimilar | Trastuzumab BS for I.V.Infusion「CTH」 | 60mg | 8,424 | 58.6 | 53.9 |
| Biosimilar | Trastuzumab BS for I.V.Infusion「CTH」 | 150mg | 19,118 | 133 | 122.4 |
| Biosimilar | TRASTUZUMAB BS FOR INTRAVENOUS DRIP INFUSION"DAIICHI SANKYO" | 60mg | 8,424 | 58.6 | 53.9 |
| Biosimilar | TRASTUZUMAB BS FOR INTRAVENOUS DRIP INFUSION"DAIICHI SANKYO" | 150mg | 19,118 | 133 | 122.4 |
| Biosimilar | TRASTUZUMAB BS for Intravenous Infusion［Pfizer］ | 60mg | 8,424 | 58.6 | 53.9 |
| Biosimilar | TRASTUZUMAB BS for Intravenous Infusion［Pfizer］ | 150mg | 19,118 | 133 | 122.4 |
|  |  |  |  |  |  |
| Reference | AVASTIN for Intravenous Infusion | 100mg | 32,305 | 225 | 206.8 |
| Reference | AVASTIN for Intravenous Infusion | 400mg | 121,608 | 846.9 | 778.3 |
| Biosimilar | BEVACIZUMAB BS Intravenous Infusion[Pfizer] | 100mg | 14,286 | 99.5 | 91.4 |
| Biosimilar | BEVACIZUMAB BS Intravenous Infusion[Pfizer] | 400mg | 54,403 | 378.9 | 348.2 |
| Biosimilar | BEVACIZUMAB BS INTRAVENOUS DRIP INFUSION"DAIICHI SANKYO" | 100mg | 14,286 | 99.5 | 91.4 |
| Biosimilar | BEVACIZUMAB BS INTRAVENOUS DRIP INFUSION"DAIICHI SANKYO" | 400mg | 54,403 | 378.9 | 348.2 |
| Biosimilar | Bevacizumab BS Intravenous Infusion | 100mg | 14,286 | 99.5 | 91.4 |
| Biosimilar | Bevacizumab BS Intravenous Infusion | 400mg | 54,403 | 378.9 | 348.2 |
| Biosimilar | Bevacizumab BS for I.V.Infusion「CTNK」 | 100mg | 14,286 | 99.5 | 91.4 |
| Biosimilar | Bevacizumab BS for I.V.Infusion「CTNK」 | 400mg | 54,403 | 378.9 | 348.2 |
| JPY: Japanese Yen, USD: US dollar, EUR: Euro | | | | | |
|  |  |  |  |  |  |
| Information on the NHI Drug Price List and Generic Drugs | |  |  |  |  |
| <https://www.mhlw.go.jp/topics/2022/04/tp20220401-01.html> | |  |  |  |  |
