# Supplementary material for: The impact of financial incentives promoting biosimilar products in oncology: A quasi-experimental study using administrative data
Source: PLoS One. 2024 Nov 14;19(11):e0312577. doi: 10.1371/journal.pone.0312577 (PMC11563361; doi:10.1371/journal.pone.0312577)
Supplement: S2 Table — (DOCX) [file pone.0312577.s002.docx]

**Supporting information**

Supplement to: Itoshima H, Takada D, Goto E, Sasaki N, Kunisawa S, Imanaka Y.

The impact of financial incentives for promotion of using biosimilar products on oncology field: A quasi-experiment design in administrative data

**Contents**

S2 Table. The ICD-10 codes used in this study

**S2 Table. The ICD-10 codes used in this study**

| Diagnosis | ICD-10 code |
| --- | --- |
| Malignant neoplasm of stomach | C16.x |
| Malignant neoplasm of colorectum | C18.x, C19.x, C20.x |
| Malignant neoplasm of trachea, bronchus and lung | C33, C34.x |
| Malignant neoplasm of breast | C50.x |
| Non-follicular lymphoma | C83.x |
| Other and unspecified types of non-Hodgkin lymphoma | C85.x |
| Malignant immunoproliferative diseases | C88.x |
| Other specified neoplasms of uncertain or unknown behaviour of lymphoid, haematopoietic and related tissue | D47.7 |
