# Supplementary material for: The impact of financial incentives promoting biosimilar products in oncology: A quasi-experimental study using administrative data
Source: PLoS One. 2024 Nov 14;19(11):e0312577. doi: 10.1371/journal.pone.0312577 (PMC11563361; doi:10.1371/journal.pone.0312577)
Supplement: S3 Table — i = hospitals. T = time period (T0, before the new health policy (financial incentive) was implemented; T1, after the new health policy was implemented); A = treatment variable (A = 0 if the unit did not receive the policy, A = 1 if the unit received the policy); A*T = the average treatment effect on the treated (interaction between the treatment indicator and the time indicator); C = set of unit-time-varying covariates that affect (and not affected by) the outcome and which represents the set of covariates sufficient for confounding control; μ = time-invariant unobserved confounders; λ = time-varying effects that are assumed to be the same for the treated and control units. X1i = intervention at hospitals (0 = no incentive [ineligible], 1 = incentive [eligible]); X2i = time indicator (0 = pre-intervention, 1 = post-intervention); Xit = a covariate that can vary across units i and time t; β0 = constant; β1 = treatment group-specific effect; β2 = time trend common to the eligible and ineligible hospitals; β3 = difference-in-differences estimates. (DOCX) [file pone.0312577.s003.docx]

**Supporting information**

Supplement to: Itoshima H, Takada D, Goto E, Sasaki N, Kunisawa S, Imanaka Y.

The impact of financial incentives for promotion of using biosimilar products on oncology field: A quasi-experiment design in administrative data

**Contents**

S3 Table. Statistical models of the generalized synthetic control method and difference-in-differences estimates

**S3 Table. Statistical models of the generalized synthetic control method and difference-in-differences estimates**

| **Model** | **Equation** |
| --- | --- |
| Generalized synthetic control method | $Y_{it =}C_{it}\beta+ \lambda_{t}\mu_{i}+ \tau_{it}AT+ \varepsilon_{it}$ |
| Difference in differences | $E\left( Y_{i} \right)=\beta_{0}+\beta_{1}X_{1i}+\beta_{2}X_{2i}+\varsigma_{t}X_{it}+\beta_{3}X_{1i}X_{2i}$ |
