# Supplementary figures and images for: The impact of financial incentives promoting biosimilar products in oncology: A quasi-experimental study using administrative data
Source: PLoS One. 2024 Nov 14;19(11):e0312577. doi: 10.1371/journal.pone.0312577 (PMC11563361; doi:10.1371/journal.pone.0312577)

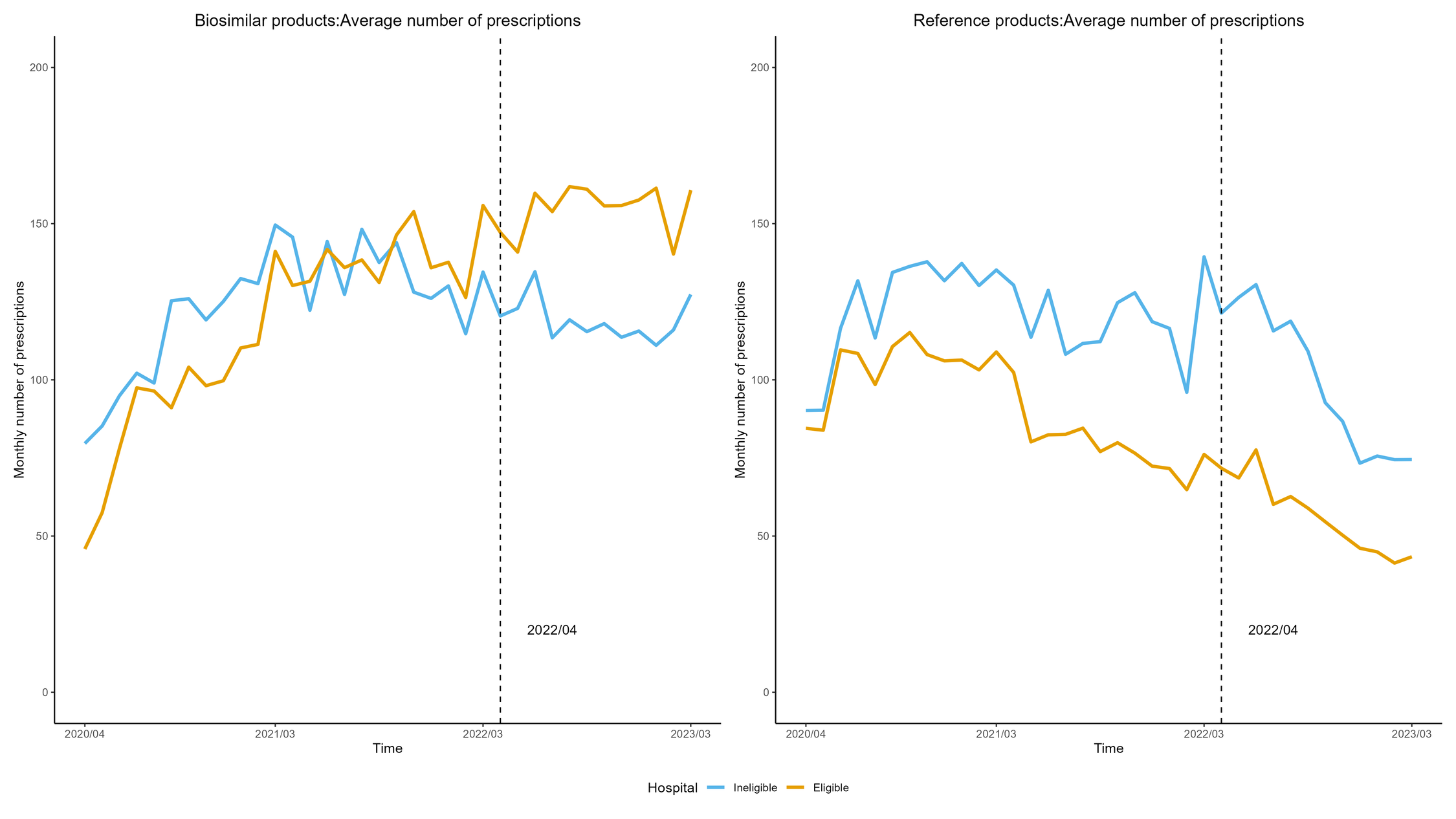

Supplement: S1 Fig — The average number of prescriptions for biosimilar (rituximab, trastuzumab, bevacizumab) and reference products by hospital. (TIF) [file pone.0312577.s004.tif]

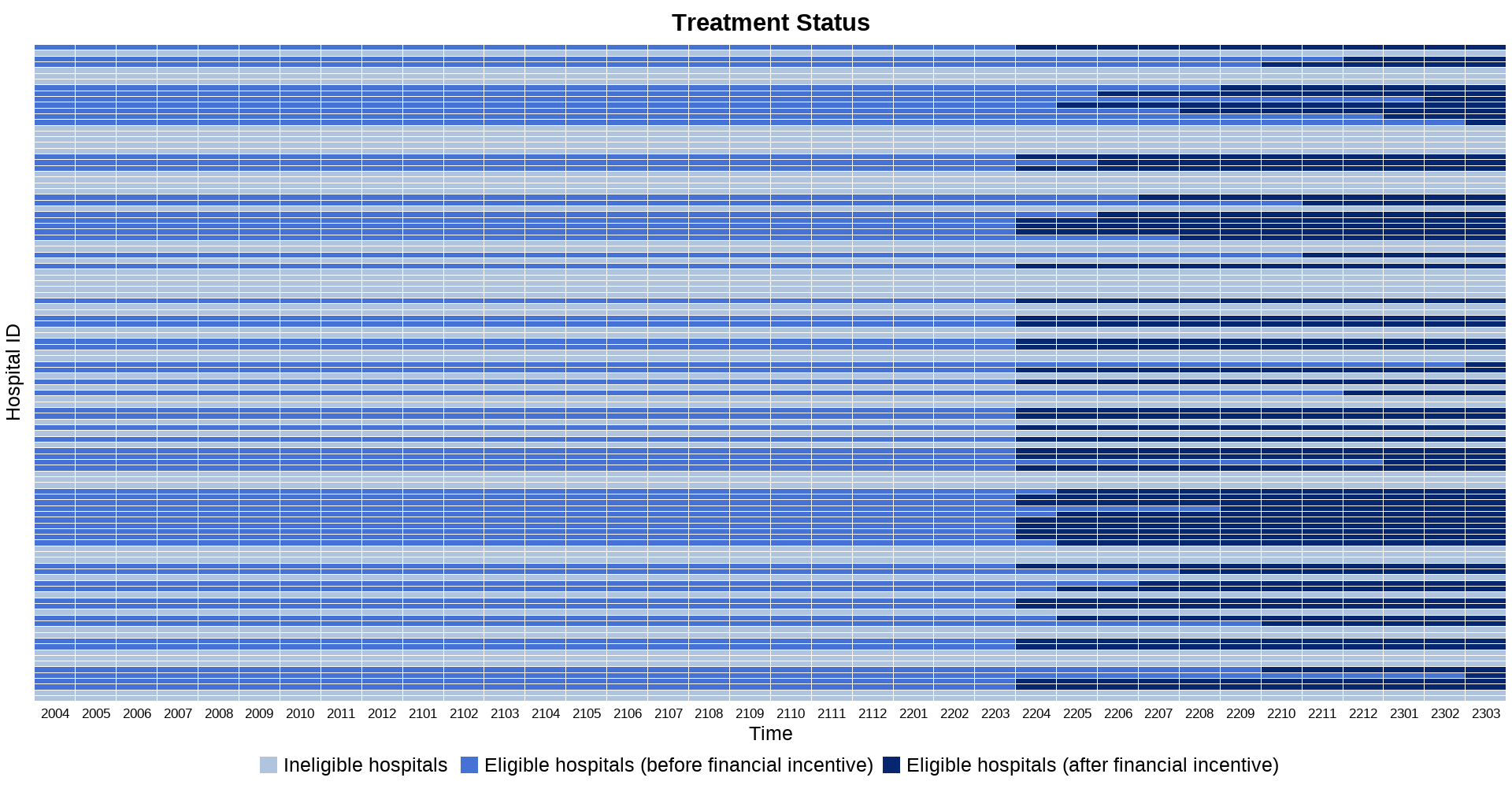

Supplement: S2 Fig — The y-axis represents each hospital, and the x-axis represents each month from April 2020 to March 2023. Light blue represents hospitals that did not receive financial incentives during the study period. Hospitals with financial incentives are represented in dark blue, while a darker blue indicates when these incentives were obtained. (TIF) [file pone.0312577.s005.tif]

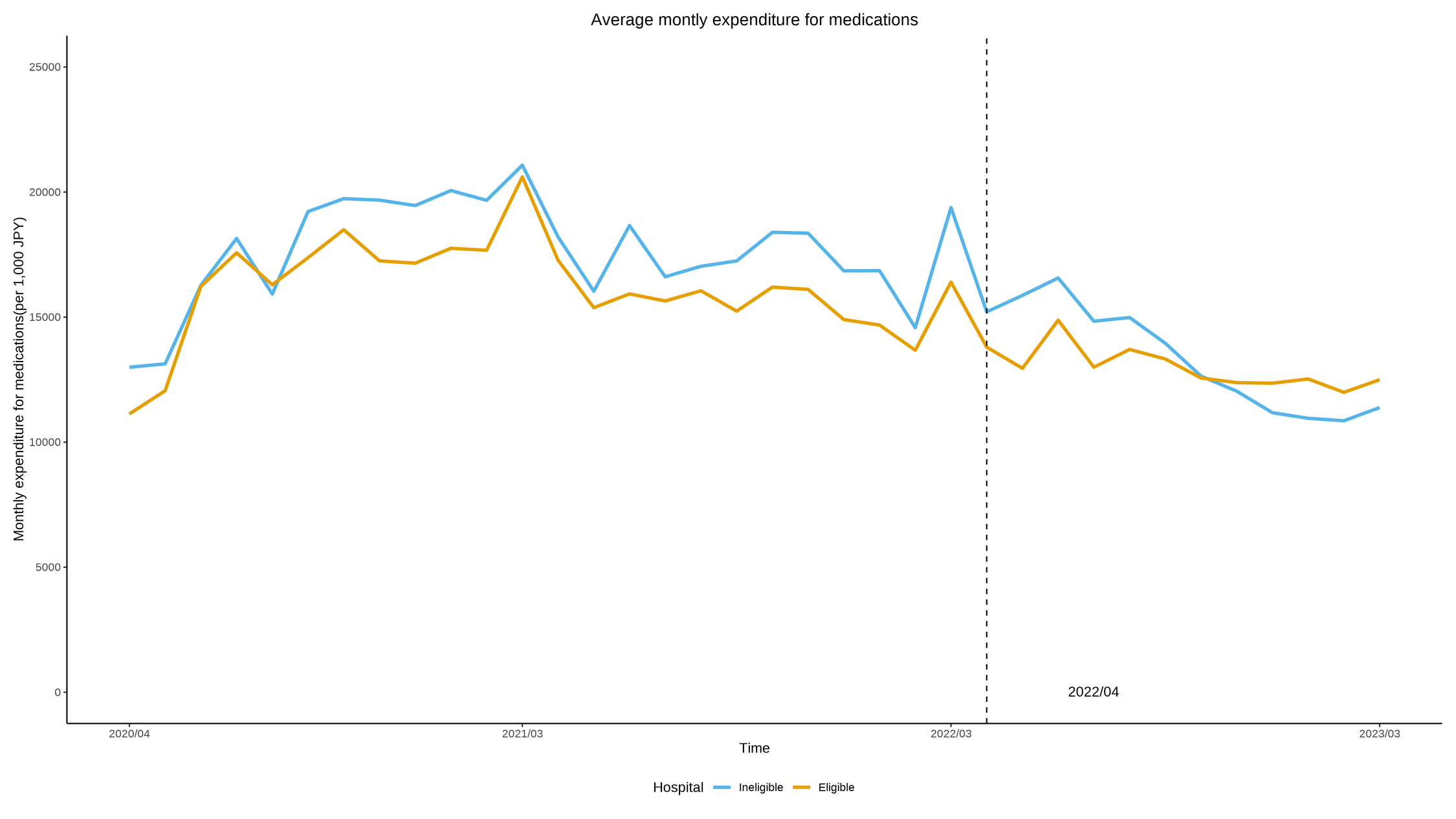

Supplement: S3 Fig — The average overall expenditure on drugs (rituximab, trastuzumab, and bevacizumab) in both eligible and ineligible hospitals. (TIF) [file pone.0312577.s006.tif]
